# Supplementary material for: Causal interplay between lactose intolerance and gut microbiota: a combined bidirectional Mendelian randomization and in vivo validation study
Source: Front Nutr. 2026 Jun 1;13:1803337. doi: 10.3389/fnut.2026.1803337 (PMC13265576; doi:10.3389/fnut.2026.1803337)
Supplement: Supplementary file 1 [file Data_Sheet_1.zip › supplementary materials/Forward/leave one out cross validation/ebi-a-GCST90027728.finngen_R12_E4_LACTONAS.pdf]

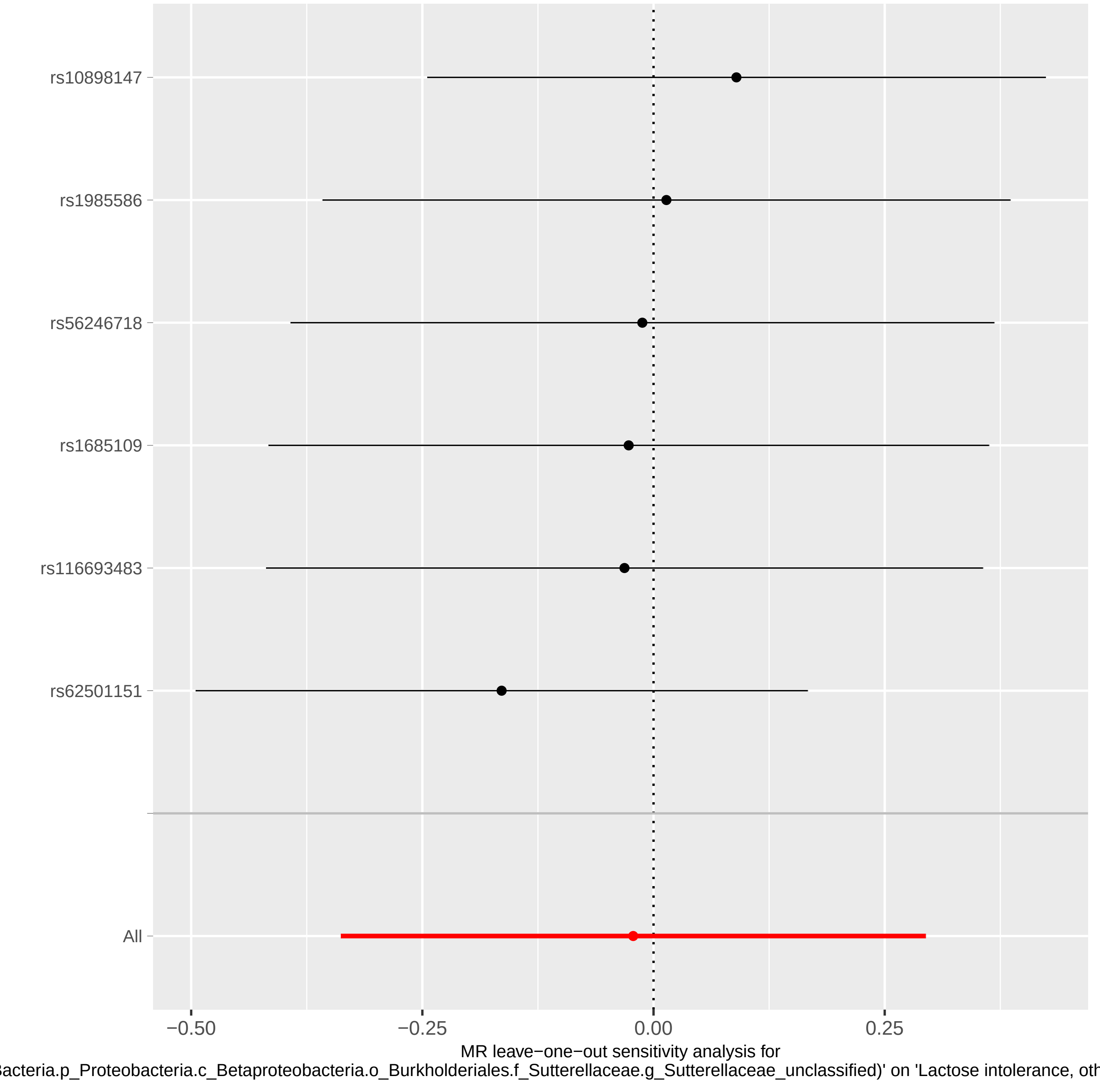
rs10898147
rs1985586
rs56246718
rs1685109
rs116693483
rs62501151
All
−0.50
−0.25
0.00
0.25
MR leave−one−out sensitivity analysis for
Bacteria.p_Proteobacteria.c_Betaproteobacteria.o_Burkholderiales.f_Sutterellaceae.g_Sutterellaceae_unclassified)' on 'Lactose intolerance, oth
